# Critical Roles of Myc-ODC Axis in the Cellular Transformation Induced by Myeloproliferative Neoplasm-Associated JAK2 V617F Mutant

**DOI:** 10.1371/journal.pone.0052844

**Published:** 2013-01-03

**Authors:** Megumi Funakoshi-Tago, Kazuya Sumi, Tadashi Kasahara, Kenji Tago

**Affiliations:** 1 Department of Biochemistry, Faculty of Pharmacology, Keio University, Tokyo, Japan; 2 Division of Structural Biochemistry, Department of Biochemistry, Jichi Medical University, Shimotsuke-shi, Japan; Emory University, United States of America

## Abstract

The acquired mutation (V617F) of Janus kinase 2 (JAK2) is observed in the majority of patients with myeloproliferative neoplasms (MPNs). In the screening of genes whose expression was induced by JAK2 (V617F), we found the significant induction of c-Myc mRNA expression mediated by STAT5 activation. Interestingly, GSK-3β was inactivated in transformed Ba/F3 cells by JAK2 (V617F), and this enhanced the protein expression of c-Myc. The enforced expression of c-Myc accelerated cell proliferation but failed to inhibit apoptotic cell death caused by growth factor deprivation; however, the inhibition of GSK-3β completely inhibited the apoptosis of cells expressing c-Myc. Strikingly, c-Myc T58A mutant exhibited higher proliferative activity in a growth-factor-independent manner; however, this mutant failed to induce apoptosis. In addition, knockdown of c-Myc significantly inhibited the proliferation of transformed cells by JAK2 (V617F), suggesting that c-Myc plays an important role in oncogenic activity of JAK2 (V617F). Furthermore, JAK2 (V617F) induced the expression of a target gene of c-Myc, ornithine decarboxylase (ODC), known as the rate-limiting enzyme in polyamine biosynthesis. An ODC inhibitor, difluoromethylornithine (DFMO), prevented the proliferation of transformed cells by JAK2 (V617F). Importantly, administration of DFMO effectively delayed tumor formation in nude mice inoculated with transformed cells by JAK2 (V617F), resulting in prolonged survival; therefore, ODC expression through c-Myc is a critical step for JAK2 (V617F)-induced transformation and DFMO could be used as effective therapy for MPNs.

## Introduction

The non-receptor tyrosine kinase, JAK2, is an essential signal transducer of various cytokine signaling, including that of erythropoietin (Epo), which is required for the proliferation and differentiation of red blood cells [1], [2]. Deregulation of the JAK2 signaling pathway promotes cell growth and prevents apoptosis in a variety of hematological malignancies, such as acute lymphoid leukemia and chronic myeloid leukemia [3], [4]. Previously, a somatic JAK2 mutation was found in a high number of myeloproliferative neoplasm (MPN) patients, that is, nearly 100% of patients with *polycythemia vera* (PV) and about 50% of patients with *essential thrombocytosis* (ET) and *primary myelofibrosis* (PMF). This mutation is a G-C to T-A transversion at nucleotide 1849 of exon 14, resulting in the substitution of valine by phenylalanine at codon 617 (V617F) [5]–[7].

Previously, we reported that the V617F mutation caused the constitutive activation of JAK2 when Epo receptor (EpoR) was coexpressed, and JAK2 (V617F) exhibited cytokine-independent survival and the proliferation of JAK2-deficient erythroid progenitor cells [8]. In addition, tumorigenesis was induced after injection of Ba/F3 cells expressing JAK2 (V617F) and EpoR into nude mice, suggesting that JAK2 (V617F) behaves as a potent oncogene product [9]. We also demonstrated that JAK2 (V617F) causes aberrant activation of a transcription factor, signal transducers and activators of transcription 5 (STAT5), which is critical for JAK2 (V617F)-induced anti-apoptotic and oncogenic activities [10].

Wernig et al. used a JAK2 mutant (V617F, Y114A), which lacks binding ability to EpoR [11]. Y114A mutation suppresses the transforming signals induced by JAK2 (V617F). These reports support the mechanism that the interaction between JAK2 (V617F) and EpoR is essential to exhibit the transforming ability of V617F mutant.

*Myc* genes (including *N-Myc*, *L-Myc* and *c-Myc*) represent a family of basic/helix-loop-helix/leucine zipper transcription factors (bHLH/LZ). The expressions of Myc genes are often overexpressed in various tumors by virtue of chromosomal amplifications, translocations, or mutations [12]–[14]. Enforced expression of Myc accelerates events related with tumorigenesis, such as cell growth, cell migration, and tumor cell metastasis [15], [16].

Ornithine decarboxylase (ODC) was identified as a target gene of Myc, and this gene product functions as a rate-limiting enzyme in polyamine biosynthesis [17]. ODC converts L-ornithine to putrescine, which is then converted into spermidine and then spermine by dedicated synthases. Polyamines are positively charged small molecules present in all living organisms and bind to and stabilize negatively charged cellular macromolecules, including nucleic acids, phospholipids, and proteins. Given their broad roles, the content of intracellular polyamine is kept under tight control in cells through transport, export, synthesis, and catabolism [18], [19]. ODC activity is frequently elevated in cancer through dysregulation of *Myc,* and this enhancement of ODC activity contributes to tumor cell proliferation [20], [21].

Our previous observations about the requirement of STAT5 for JAK2 (V617F)-induced tumorigenesis have pointed out the possibility that STAT5-targeted gene expression could play the central role in oncogenic activity of JAK2 (V617F), and this is most likely to be the mechanism of how MPNs are caused by JAK2 (V617F). In the current study, we focused on the alteration of gene expression, which is caused by the JAK2 (V617F)-induced signaling pathway, especially mediated by STAT5. We found that JAK2 (V617F) induced constitutive expression of c-Myc and one of its target genes, ODC. Moreover, we showed that an ODC inhibitor, α-difluoromethylornithine (DFMO), significantly abrogated the proliferation of transformed BaF3 cells by JAK2 (V617F) *in vitro* and efficiently inhibited JAK2 (V617F)-induced tumor formation in nude mice. Together, these data strongly support that ODC expression induced by c-Myc is critical for JAK2 (V617F)-driven transformation and that targeted disruption of the c-Myc-ODC axis may have therapeutic utility for the treatment of MPNs.

## Experimental Procedures

### Reagents

Recombinant human erythropoietin (Epo) (ESPO 3000) and recombinant murine IL-3 were purchased from Kirin Brewery Co. (Tokyo, Japan) and PEPROTECH (Rocky Hill, NJ, USA), respectively. AG490 and DL-α-difluoromethylornithine (DFMO) were purchased from TOCRIS Bioscience (Ellisville, MO, USA). GSK-3β inhibitor II was purchased from Calbiochem (San Diego, CA, USA). Spermidine and anti-Flag antibody (M2) were purchased from Sigma-Aldrich (St. Louis, MO, USA). Anti-JAK2 antibody (Y1007/1008), anti-phospho-STAT5 antibody (Y694), anti-STAT5 antibody, anti-phospho-GSK-3β antibody (S9), anti-phospho-Rb antibody (S801/811) and anti-p27 antibody were purchased from Cell Signaling Technology (Danvers, MA, USA). Anti-phospho-c-Myc (T58) and anti-HA antibody (3F10) were purchased from Abcam Inc. (Cambridge, MA, USA) and Roche Applied Science (Indianapolis, IN, USA), respectively. Anti-β-actin antibody, anti-c-Myc antibody (N262), anti-EpoR antibody and anti-ODC antibody were purchased from Santa Cruz Biotechnology Inc. (Santa Cruz, CA, USA). Peroxidase-conjugated secondary antibodies were from Dako (Glostrup, Denmark).

### Plasmids

Murine JAK2-HA and murine EpoR c-Flag were subcloned into retroviral plasmids, murine stem cell virus (MSCV)-Hygro and MSCV-Puro (Clontech, CA, USA), respectively [10]. Mutagenesis of amino acid residue, V617F in JAK2 and T58A in c-Myc was performed using a site-directed mutagenesis kit (Stratagene, La Jolla, CA, USA). Murine c-Myc and mutants of c-Myc (T58A, In373) were subcloned into MSCV-Puro. The sequences of oligonucleotides used for constructing shRNA retroviral vector were as follows: sh-STAT5∶5′-gatccccgcggcgagagatcctgaacaattcaagagattgttcaggatctctcgccgcttttta-3′ and 5′-agcttaaaaagcggcgagagatcctgaacaatctcttgaattgttcaggatctctcgccgcggg-3′, sh-c-Myc: 5′-gatccccgcgacgaggaagagaatttttcaagagaaaattctcttcctcgtcgcttttta-3′ and 5′-agcttaaaaagcgacgaggaagagaattttctcttgaaaaattctcttcctcgtcgcggg-3′. Underlined sequences correspond to the sequence of murine STAT5 (from 1281 to 1299 in ORF), and c-Myc (from 617 to 715 in ORF), respectively.

### Retrovirus Infection and Cell Cultures

Ba/F3 cells were infected with empty virus (-), wild-type murine JAK2 c-HA, or a mutant of murine JAK2 c-HA (V617F) with murine EpoR c-FLAG as described previously [8]–[10]. Ba/F3 cells were infected with empty virus (-) and retroviruses encoding wild-type murine c-Myc, T58A mutant and In373 mutant of c-Myc. These cells were cultured in RPMI 1640 medium (Nacalai Tesque, Tokyo, Japan) supplemented with 10% fetal bovine serum (BioWest, France), 100 units/ml penicillin (Nacalai Tesque), 100 µg/ml streptomycin (Nacalai Tesque), with Epo (5 U/mL) or IL-3 (2 ng/mL). To select infected cells, 5 µg/mL puromycin and 200 µg/mL hygromycin were used. All the cells utilized in the current study and the genes of their infected retroviruses are described in Table 1. HEL cells were cultured in RPMI 1640 medium supplemented with 10% fetal bovine serum, 100 units/ml penicillin and 100 µg/ml streptomycin.

**Table 1:** List of Ba/F3-derived cell lines analyzed in this study.

Cell’s name	Infected retroviruses					c-Myc expression		Tumori-genesis	Fig
	EpoR	JAK2	STAT5	c-Myc^a^	shRNA	mRNA	Protein		
Control	−	−	−	−	−	ND^c^	ND^c^	ND^c^	
EpoR	WT	−	−	−	−	ND^c^	ND^c^	ND^c^	1,3,6,7
WT/EpoR	WT	WT	−	−	−	ND^c^	ND^c^	ND^c^	1,3,6,7
VF/EpoR	WT	V617F	−	−	−	++	++	+++	1,3,6,7
VF/Y479F	Y479F	V617F	−	−	−	++	ND^c^	Not Tested	3
Ba/F3-Stat5	−	−	WT	−	−	ND^c^	ND^c^	ND^c^	1
Ba/F3-1*6	−	−	Active	−	−	++	++	+++ (ref10)	1
Ba/F3-Myc	−	−	−	WT	−			+	2,3,4
Ba/F3-T58A	−	−	−	T58A	−			+++	2,3,4
Ba/F3-In373	−	−	−	In373	−			ND^c^	2,3,4
VF/EpoR/sh-control	WT	V617F	−	−	Luc	++	++	+++	1,5
VF/EpoR/sh-STAT5	WT	V617F	−	−	STAT5	ND^c^	ND^c^	ND^c^	1
VF/EpoR/sh-c-Myc	WT	V617F	−	−	c-Myc	ND^c^	ND^c^	ND^c^	5

a Fig. 2A shows structures of c-Myc mutants, T58A and In373.

b c-Myc expression was tested under the absence of Epo stimulation.

c ND means “Not Detected”.

d Tumorigenesis was tested by the transplantation of cells into nude mice.

### DNA Microarray Analysis

Total RNAs were prepared from Ba/F3 cells expressing wild-type JAK2 and EpoR (WT/EpoR cells) and Ba/F3 cells expressing JAK2 V617F mutant and EpoR (V617F/EpoR cells) cultured without Epo for 12 hr. DNA microarray (TORAY, 3D-Gene, Mouse Oligo chip 24k) was performed and the enhanced gene expression induced by JAK2 V617F mutant was determined. The microarray data were registered with NCBI’s Gene Expression Omnibus (GEO) database under accession number GSE34239.

### Ba/F3 Cell Growth Assay

Transduced and exponentially growing Ba/F3 cells were washed twice with phosphate-buffered saline (PBS) and incubated with RPMI 1640 medium supplemented with 10% fetal bovine serum for 24 hr. Cell viability was checked by the trypan blue exclusion method [9].

### Cell Cycle Analysis

Cells were fixed with 70% (v/v) ethanol at −20°C overnight. Cells were then centrifuged at 2,000×g for 2 min and resuspended in PBS containing 10 µg/ml RNase A (Wako, Tokyo, Japan) and 100 µg/ml propidium iodide (PI) (Sigma). Following 30 min incubation, cell cycle parameters were determined by flow cytometry analysis using FACSCalibur [10]. All data were recorded and analyzed using CellQuest software.

### DNA Fragmentation Assay

Genomic DNA was prepared for gel electrophoresis as described previously [10]. Electrophoresis was performed on a 1% (w/v) agarose gel in Tris/boric acid buffer. Fragmented DNA was visualized by staining with ethidium bromide after electrophoresis.

### Immunoblotting

Cell lysates were prepared with Nonidet P-40 lysis buffer (50 mM Tris-HCl, pH 7.4, 10% glycerol, 50 mM NaCl, 0.5% sodium deoxycholate, 1% Nonidet P-40, 20 mM NaF, 0.2 mM Na_3_VO_4_) supplemented with protease inhibitors. Denatured samples were run on 10% SDS-PAGE and transferred to PVDF membranes. Immunoblotting was performed as previously described [8].

### RNA Isolation and Reverse Transcription-Polymerase Chain Reaction (RT-PCR)

RNA was prepared using an RNA purification kit (Qiagen, Hilden, Germany). RT was performed using an oligo (dT)_20_ primer and 2 µg total RNA for first-strand cDNA synthesis, as described previously [10]. Quantitative real-time PCR was performed using an ABI Prism 7900HT Sequence detection system (Applied Biosystems, Scoresby, Victoria, Australia). PCR primer sequences were as follows: c-Myc 5′-gctcggtctactctggcatc-3′(upstream) and 5′-tgttctcgtccttgatgtcg-3′ (downstream); Pim-2 5′-actcagtcacctgcccactt-3′ (upstream) and 5′-ccggaaccaaaatcaatgag-3′ (downstream); Bcl-XL 5′-tggtggtcgactttctctcc-3′(upstream) and 5′-ctccatcccgaaagagttca-3′ (downstream); GAPDH, 5′-actccactcacggcaaattc-3′ (upstream) and 5′-ccttccacaatgccaaagtt-3′ (downstream); ectopic c-Myc 5′-cccttgaacctcctcgttcgacc-3′ (MSCV-5′) and 5′-tgttctcgtccttgatgtcg-3′ (downstream).

### Tumor Transplantation into Nude Mice and Administration of DFMO

BALB/c nude mice aged 4 weeks were injected subcutaneously (s.c.) with 1 × 10^7^ transduced Ba/F3 cells. After injection of empty virus-infected cells and V617F/EpoR cells, the mice were given either water or water containing 1% DFMO for 10 days. Sixteen days post-inoculation of transduced cells, the animals were sacrificed, and liver and spleen weights were recorded.

### Histological Examination

The liver was fixed in 4% paraformaldehyde, then dehydrated gradually in alcohol, embedded in paraffin and sectioned at a thickness of 2 µm. The sections were stained with hematoxylin and eosin and analyzed for the presence of tumor cell infiltration using an Olympus BX50 microscope (Olympus, Tokyo, Japan) with Olympus Micro DP70 software.

## Results

### JAK2 (V617F) Induces c-Myc Expression Through the Activation of STAT5

**Figure 1:**
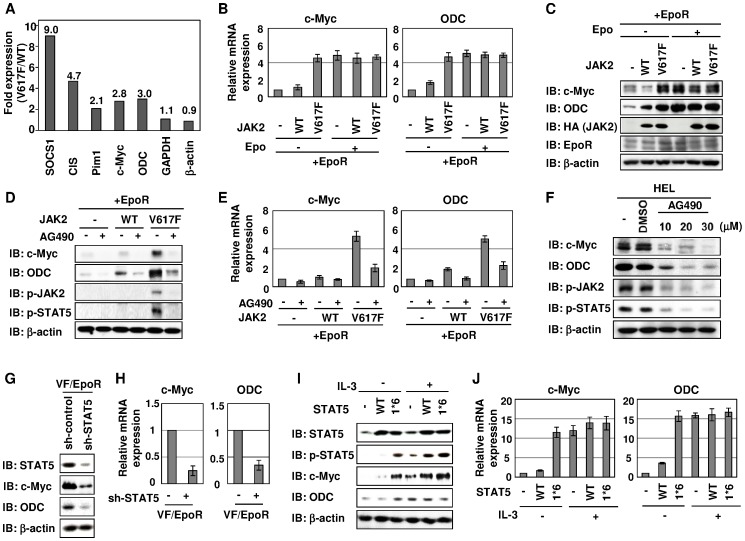
JAK2 (V617F) induced expression of c-Myc through STAT5 activation. (A–E) Ba/F3 cell lines were infected with empty virus (−), retroviruses encoding wild-type JAK2 c-HA (WT), JAK2 mutant c-HA (V617F) and EpoR c-Flag as indicated in each figure. (A) WT/EpoR cells and VF/EpoR cells were cultured without Epo for 12 hr, and then total RNAs were prepared from each cell line. The enhancement of gene expression induced by JAK2 (V617F) was determined by DNA array. The ratio of the altered gene expressions in VF/EpoR cells was calculated by dividing the amount of each gene in VF/EpoR cells by their amount in WT/EpoR cells. (B, C) A series of Ba/F3 cells expressing the described genes was cultured untreated or stimulated with Epo (5 U/mL) for 12 hr. (D, E) A series of Ba/F3 cells expressing the indicated genes were cultured in the absence of Epo, and treated with DMSO (0.1%) or AG490 (30 µM) for 12 hr. (F) HEL cells were treated with DMSO (0.1%) or AG490 (10, 20, 30 µM) for 24 hr. (G, H) VF/EpoR cells were infected with retrovirus harboring shRNA against firefly luciferase (control) or STAT5. (I, J) Ba/F3 cells were infected with empty virus (−), retrovirus encoding wild-type STAT5 (WT) or the constitutively active mutant of STAT5 (1*6). The cells were cultured without Epo for 12 hr. (B, E, H, J) The mRNA expression of c-Myc and ODC was analyzed by quantitative real-time PCR. GAPDH mRNA was analyzed as an internal control. Data are the mean ± S.D. of the relative expression levels in three experiments. (C, D, F, G, I) Whole cell lysates were immunoblotted (IB) with anti-c-Myc antibody, anti-ODC antibody, anti-HA antibody, anti-EpoR antibody, anti-phospho-JAK2 antibody (Y1007/1008), anti-phospho-STAT5 antibody (Y694), anti-STAT5 antibody or anti-β-actin antibody.

Previously, we reported that constitutive activation of STAT5 induced by JAK2 (V617F) conferred growth-factor independence on Ba/F3 cells [10] and assumed that a wide range of genes induced by STAT5 activation seems to play important roles in cellular transformation induced by JAK2 (V617F); therefore, by performing DNA array analysis, the alteration of the gene expression was examined between cells expressing wild-type JAK2 with EpoR (WT/EpoR cells) and cells expressing JAK2 (V617F) with EpoR (VF/EpoR cells). As shown in Fig. 1A, several genes such as SOCS1, CIS and Pim1, which are known as direct target genes of STAT5, were upregulated in VF/EpoR cells [22]–[24]. Compared with WT/EpoR cells, the mRNA expressions of a transcription factor, c-Myc, and its target gene, ODC, in VF/EpoR cells were upregulated by about three times (Fig. 1A). To assess this, mRNA and protein levels of c-Myc and ODC in WT/EpoR cells and VF/EpoR cells were examined by RT-PCR and immunoblotting, respectively. In cells expressing EpoR (EpoR cells) and WT/EpoR cells, Epo stimulation significantly induced the expression of c-Myc mRNA. In contrast, in VF/EpoR cells, high expression of c-Myc mRNA was observed regardless of Epo stimulation. Furthermore, c-Myc protein levels were markedly elevated in VF/EpoR cells in the absence of Epo stimulation (Fig. 1B, C). Also in the absence of Epo stimulation, the expression of ODC mRNA was hardly detected in EpoR cells, and this was slightly enhanced by the ectopic expression of wild-type JAK2; however, the expression of ODC mRNA was markedly elevated in VF/EpoR cells even to the same level as when cells were stimulated with Epo (Fig. 1B). Moreover, it was confirmed that the expression of ODC protein was also up-regulated in cells expressing JAK2 V617F mutant regardless of Epo stimulation (Fig. 1C). Strikingly, JAK2 inhibitor AG490 significantly suppressed not only the phosphorylation of JAK2 and STAT5 but also the expression of c-Myc and ODC induced by JAK2 (V617F) (Fig. 1D, E). In addition, in human erythroleukemia (HEL) cells, which are homozygous for the V617F mutation in JAK2 [25], high expression of c-Myc and ODC was observed, and treatment with AG490 diminished the phosphorylation of JAK2 and STAT5, and also the expression of c-Myc and ODC (Fig. 1F). Interestingly, shRNA against STAT5 (sh-STAT5) effectively decreased the STAT5 expression, and reduced the expression of c-Myc and ODC at both mRNA and protein levels in VF/EpoR cells (Fig. 1G, H). Next, we tested whether STAT5 harbors the ability to induce the expression of c-Myc. As shown in Fig. 1I and 1J, IL-3 stimulation induced the expression of c-Myc, and this was potentiated by the enforced expression of both wild-type and constitutive active STAT5 mutant (1*6). Furthermore, the expression of a constitutive active STAT5 mutant (1*6) induced the expression of c-Myc and ODC in Ba/F3 cells in the absence of IL-3 stimulation. It is also striking that the expression level of ODC mRNA exhibited a similar alteration. These results indicate that the expression of c-Myc is regulated by STAT5 activation.

### Unphosphorylated c-Myc at Thr58 Causes the Transformation of Ba/F3 Cells

**Figure 2:**
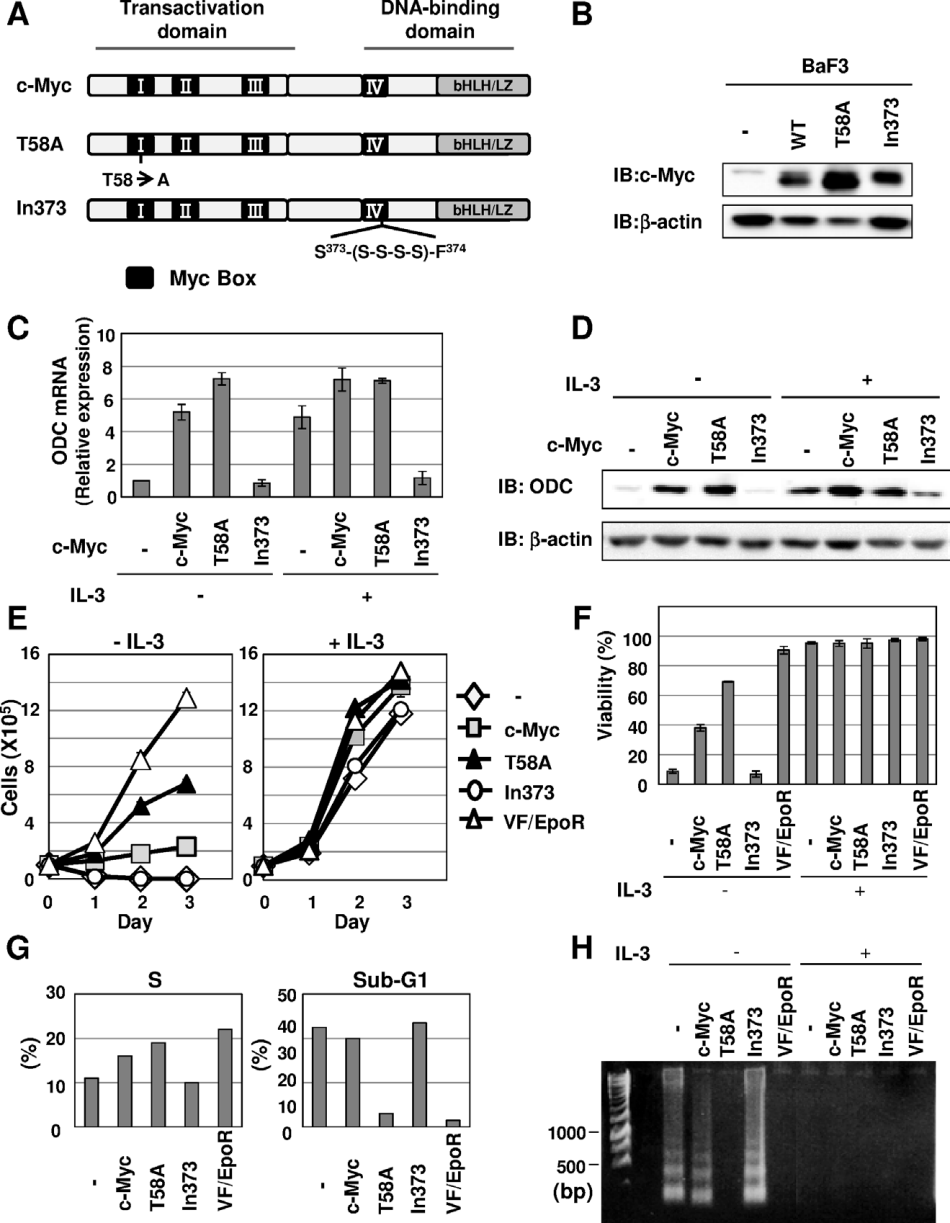
c-Myc T58A mutant conferred growth-factor independence on Ba/F3 cells. (A) Schematic diagram of wild-type (c-Myc), T58A and In373 mutants of c-Myc. (B-H) Ba/F3 cells were infected with empty virus (−) or retrovirus encoding wild-type c-Myc (c-Myc) or two c-Myc mutants (T58A, In373). (B) Transduced cells were incubated without IL-3 (2 ng/mL) for 6 hr and whole cell lysates were immunoblotted (IB) with anti-c-Myc antibody or anti-β-actin antibody. (C, D) Transduced Ba/F3 cells were cultured with/without IL-3 (2 ng/mL) for 12 hr. (C) ODC mRNA was analyzed by quantitative real-time PCR. GAPDH mRNA was analyzed as an internal control. Data are the mean ± S.D. of the relative expression levels in three independent experiments. (D) Whole cell lysates were immunoblotted (IB) with anti-ODC antibody or anti-β-actin antibody. (E) Viable transduced Ba/F3 cells and VF/EpoR cells were counted in the presence and absence of IL-3 (2 ng/mL) for 3 days. Data are the mean ± S.D. of the relative expression levels in three independent experiments. (F) Transduced Ba/F3 cells were left untreated or stimulated with IL-3 (2 ng/mL) for 24 hr. The viability of these cells was determined by the trypan blue exclusion method. Results are the mean ± S.D. of three independent experiments. (G) Transduced cells were cultured without IL-3 (2 ng/mL) for 24 hr. Cells were then fixed, treated with propidium iodide (PI) and subjected to FACS analysis. (H) Transduced Ba/F3 cells were cultured with/without IL-3 (2 ng/mL) for 24 hr. DNA was isolated from cells and subjected to agarose gel electrophoresis.

To investigate the roles of c-Myc in the JAK2 (V617F)-mediated signaling pathway, we established Ba/F3 cells infected with empty virus (Control cells), or viruses encoding wild-type c-Myc and two kinds of c-Myc mutants. c-Myc is phosphorylated at Thr58 within the Myc Box 1 region located in the N-terminal transactivation domain and phosphorylation of Thr58 targets c-Myc for ubiquitination and degradation [26], [27]. Then, we generated a point mutant in which Thr58 is substituted for alanine (T58A) in c-Myc. We also constructed an inactive mutant of c-Myc (In373), which carries an insertion in the DNA-interacting region and fails to bind to DNA, as shown in Fig. 2A [28]. As reported previously [26], [27], the expression level of T58A mutant was markedly higher than wild-type c-Myc and In373 mutant (Fig. 2B). Next, to examine the activity of c-Myc mutants, the expression level of ODC mRNA in Ba/F3 cells expressing c-Myc, T58A, and In373 (called Ba/F3-Myc, -T58A, and -In373 cells, respectively) was examined by RT-PCR analysis. Expectedly, in the absence of IL-3, both c-Myc and T58A mutant induced marked expression of ODC at both mRNA and protein levels, while In373 mutant did not. Stimulation with IL-3 induced the expressions of ODC mRNA and protein, and these were effectively enhanced by the enforced expression of wild-type c-Myc and T58A mutant, while In373 mutant markedly suppressed them. These observations support that the induction of ODC expression requires the transcriptional activity of c-Myc (Fig. 2C, D). Next, to test the effect of c-Myc on cell proliferation, the number of viable cells was counted in the absence or presence of IL-3. In the presence of IL-3, all cell types exhibited marked viability and cell proliferation. In particular, wild-type c-Myc and T58A enhanced the cell proliferation rate by about 1.5 times compared to the control cells and cells expressing In373 mutant, and similar enhancement of cell proliferation was also observed in the case of VF/EpoR cells. On the other hand, in the absence of IL-3, only Ba/F3-T58A cells survived and exhibited vigorous proliferative activity; however, its proliferation rate was lower than the proliferation rate of VF/EpoR cell. In addition, wild-type c-Myc showed slight enhancement of the viable cell number and cell proliferation. In contrast, control cells and Ba/F3-In373 cells reduced the number of viable cells, suggesting that DNA binding activity of c-Myc is required for the cell survival and proliferation induced by c-Myc (Fig. 2E). As shown in Fig. 2F, the cell viability of each cell was also tested. After withdrawing IL-3, the viability of cells expressing T58A mutant was about 70% and a similar effect was observed when VF/EpoR cells were analyzed. On the other hand, the cells expressing wild-type c-Myc exhibited about 40% cell viability; however, neither control cells nor Ba/F3-In373 cells resulted in cell viability of only 10% or less. Treatment with IL-3 enhanced the viability of all types of cells to almost 90%. We next analyzed the cell cycle distribution of these cells following 12 hr of IL-3 deprivation. Compared to control cells and cells expressing In373 mutant, the expression of wild-type c-Myc and T58A mutant increased the percentage of cells in the S phase as well as VF/EpoR cells, suggesting that both c-Myc and T58A mutant exhibited the tendency to accelerate cell proliferation. In contrast, c-Myc is also reported to cause apoptotic cell death when its expression is enforced [15]; therefore, we next analyzed the accumulation of sub-G_1_ phase induced by c-Myc and its mutants. Only T58A mutant prevented accumulation in the sub-G_1_ phase caused by growth factor deprivation, while the sub-G1 phase was increased up to more than 40% in control cells and Ba/F3-Myc and Ba/F3–In373 cells (Fig. 2G). On the other hand, we observed little accumulation of VF/EpoR cells in the sub-G_1_ phase, similar to the T58A mutant. Furthermore, DNA internucleosomal fragmentation was clearly detected in these cells following 12 hr IL-3 deprivation, except for cells expressing T58A and VF/EpoR cells, confirming that these cells underwent apoptotic cell death (Fig. 2H). These observations indicated that the expression of T58A mutant conferred growth factor independence on Ba/F3 cells, and strongly suggested that preventing phosphorylation at Thr58 accelerated c-Myc-mediated cell proliferation and anti-apoptotic activities.

### JAK2 (V617F) Enhances the Protein Stability of c-Myc Through the Inhibition of GSK-3β

**Figure 3:**
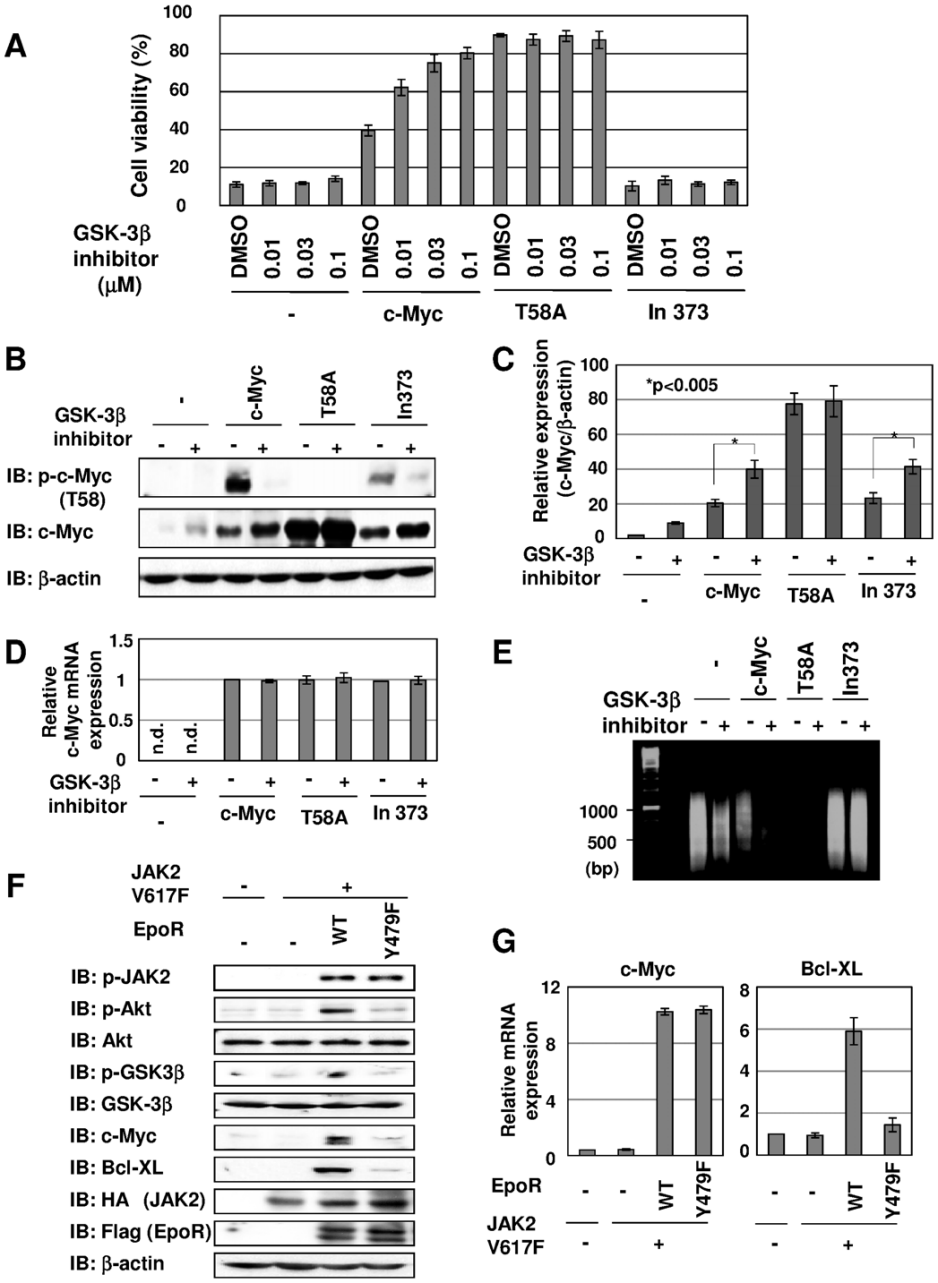
Inhibition of GSK-3β inhibited downregulation of c-Myc and apoptosis. (A) Transduced Ba/F3 cells were treated with DMSO (0.1%) or the indicated concentrations of GSK-3β inhibitor in the absence of IL-3 for 18 hr. The viability of these cells was determined by the trypan blue exclusion method. Results are the mean ± S.D. of three independent experiments. (B-E) Transduced Ba/F3 cells were treated with DMSO (0.1%) or GSK-3β inhibitor (0.1 µM) for 18 hr. (B) Whole cell lysates were immunoblotted (IB) with anti-phospho-c-Myc antibody (Thr58), anti-c-Myc antibody or anti-β-actin antibody. (C) The expression amounts of c-Myc and its mutants were normalized with the protein amount of β-actin, and the quantified ratios of c-Myc and its mutants (T58A, In373) are shown in the graph. Results are the mean ± S.D. of three independent experiments. (D) Total RNA was prepared and mRNA of ectopic c-Myc and its mutants (T58A, In373) was detected by quantitative real time-PCR. GAPDH mRNA was analyzed as an internal control. Data are the mean ± S.D. of the relative expression levels in three independent experiments. (E) DNA was isolated from cells and subjected to agarose gel electrophoresis. (F, G) Ba/F3 cell lines were infected with empty virus (−), retrovirus encoding JAK2 mutant c-HA (V617F) and retroviruses encoding wild-type EpoR c-Flag (WT) or EpoR mutant c-Flag (Y479F). Transduced Ba/F3 cells were incubated without IL-3 for 12 hr. (F) Whole cell lysates were immunoblotted (IB) with anti-phospho-JAK2 antibody (Y1007/1008), anti-phospho-GSK-3β antibody (S9), anti-GSK-3β antibody, anti-c-Myc antibody, anti-Bcl-XL antibody, anti-HA antibody, anti-Flag antibody or anti-β-actin antibody. (G) c-Myc mRNA and Bcl-XL mRNA were analyzed by quantitative real-time PCR. GAPDH mRNA was evaluated as an internal control. Data are the mean ± S.D. of the relative expression levels in three independent experiments.

It has been reported that the phosphorylation of c-Myc at Thr58 is catalyzed by GSK-3β [26], [27]; therefore, we next examined whether the regulation of GSK-3β affects c-Myc-induced apoptosis. We added the GSK-3β inhibitor to the culture of control Ba/F3 cells and cells expressing wild-type c-Myc and its mutants (T58A and In373), and tested the effect of GSK-3β inhibitor on cell death induced by cytokine withdrawal. As shown in Fig. 3A, GSK-3β inhibitor increased the viability of Ba/F3 cells expressing c-Myc in a dose-dependent manner, whereas the viability of control cells and cells expressing In373 mutant was not affected by GSK-3β inhibitor (Fig. 3A). Strikingly, Ba/F3-T58A cells exhibited high viability without treatment with the inhibitor, and their viability was comparable to Ba/F3-Myc cells treated with GSK-3β inhibitor. As shown in Fig. 3B, ectopic c-Myc and In373 mutant were phosphorylated at Thr58, and GSK-3β inhibitor completely suppressed their phosphorylation. Treatment with GSK-3β inhibitor markedly enhanced the expression of c-Myc and In373 mutant proteins (Fig. 3B; compare lanes 3 to 4, and 7 to 8, respectively). On the other hand, T58A mutant exhibited much higher expression without GSK-3β inhibitor, and its expression was not affected by the inhibitor, suggesting that the GSK-3β inhibitor-induced enhancement of c-Myc proteins is due to inhibition of the phosphorylation of c-Myc at Thr58. Indeed, the expression of endogenous c-Myc was also induced by GSK-3β inhibitor in control Ba/F3 cells (Fig. 3B, C). On the other hand, the mRNA expression level of ectopic c-Myc and its mutants was not elevated by treatment with GSK-3β inhibitor (Fig. 3D). Furthermore, when treated with GSK-3β inhibitor, DNA fragmentation was not observed in cells expressing c-Myc, and this is well reflected in the results shown in Fig. 3A (Fig. 3E). Previously, we demonstrated that phosphorylation at Tyr479 in EpoR is essential for JAK2 V617F mutant-induced Akt activation [29]. It is also known that Akt phosphorylates GSK-3β, which negatively regulates the activity of GSK-3β [30]. We next tested the requirement of Akt activation for the regulation of GSK-3β activity in the process of JAK2 (V617F)-induced transformation. As shown in Fig. 3F, the coexpression of JAK2 (V617F) and EpoR induced the phosphorylation of Akt and GSK-3β, whereas JAK2 (V617F) alone failed to cause the phosphorylation of Akt and GSK-3β. Interestingly, when EpoR mutant (Y479F) was coexpressed, the phosphorylation of Akt and GSK-3β was not induced by JAK2 (V617F) (Fig. 3F, see lane 4). These observations well fit the results of the expressions of c-Myc; however, the expression of c-Myc mRNA was not altered by the expression of JAK2 (V617F) with EpoR or EpoR (Y479F) (Fig. 3G), suggesting that the downregulation of c-Myc observed in VF/Y479F is caused by post-translational processes. Anti-apoptotic protein, Bcl-XL, was also reported to be induced by JAK2 (V617F) through Akt activation [29]. Unlike c-Myc, the induction of Bcl-XL was significantly reduced at both mRNA and protein levels in VF/Y479F cells (Fig. 3F, G). Therefore, our observations strongly suggested that JAK2 (V617F) enhanced the expression of c-Myc by not only transcription but also stabilization of c-Myc protein mediated by Akt-induced inhibition of GSK-3β.

### c-Myc Exhibits Moderate Transforming Activity, Although not Like JAK2 (V617F)

**Figure 4:**
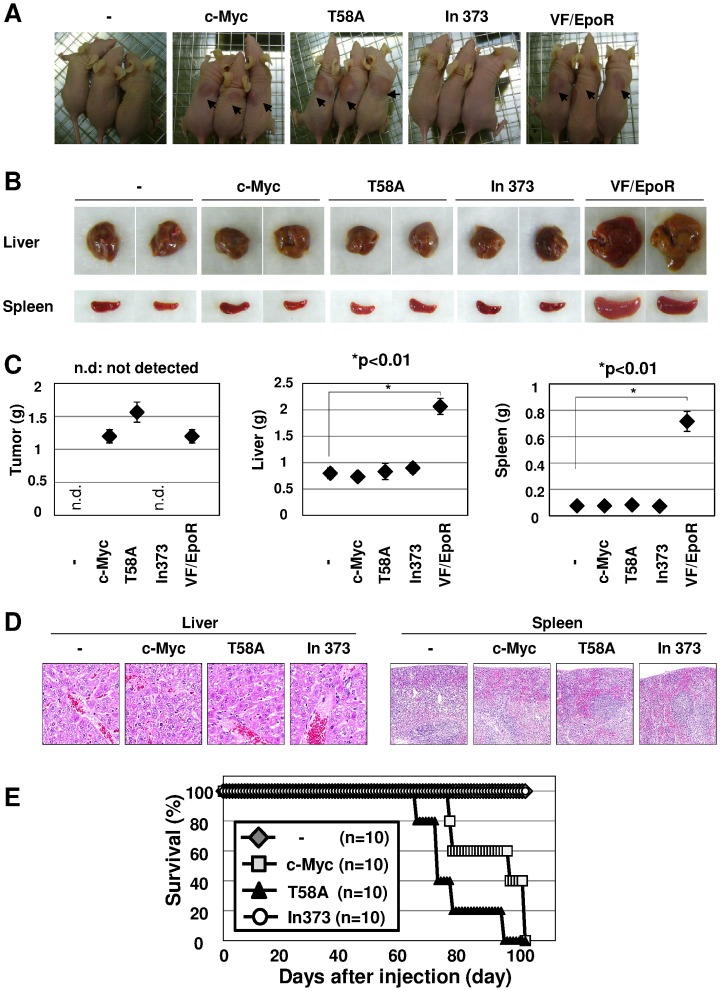
c-Myc T58A mutant significantly induced tumor formation in nude mice. Ba/F3 cells were infected with empty virus (-) and retrovirus encoding wild-type c-Myc (c-Myc) or two c-Myc mutants (T58A, In373). Transduced Ba/F3 cells and VF/EpoR cells were s.c. injected into nude mice (1×10^7^ cells/mice). (A) Nude mice were photographed 18 days post-inoculation. Arrows indicate tumors in nude mice. (B, C) Eighteen days post-inoculation, three mice were sacrificed. The morphology of the liver and spleen were photographed. The weights of the spleen and liver were measured and shown in the graphs. * indicates significant difference p<0.01 (n = 3). (D) Sixty days after inoculation, sections of the spleen and liver were stained with hematoxylin and eosin (magnification: ×100). (E) Mouse survival was monitored daily for 100 days post-inoculation (n = 10).

**Figure 5:**
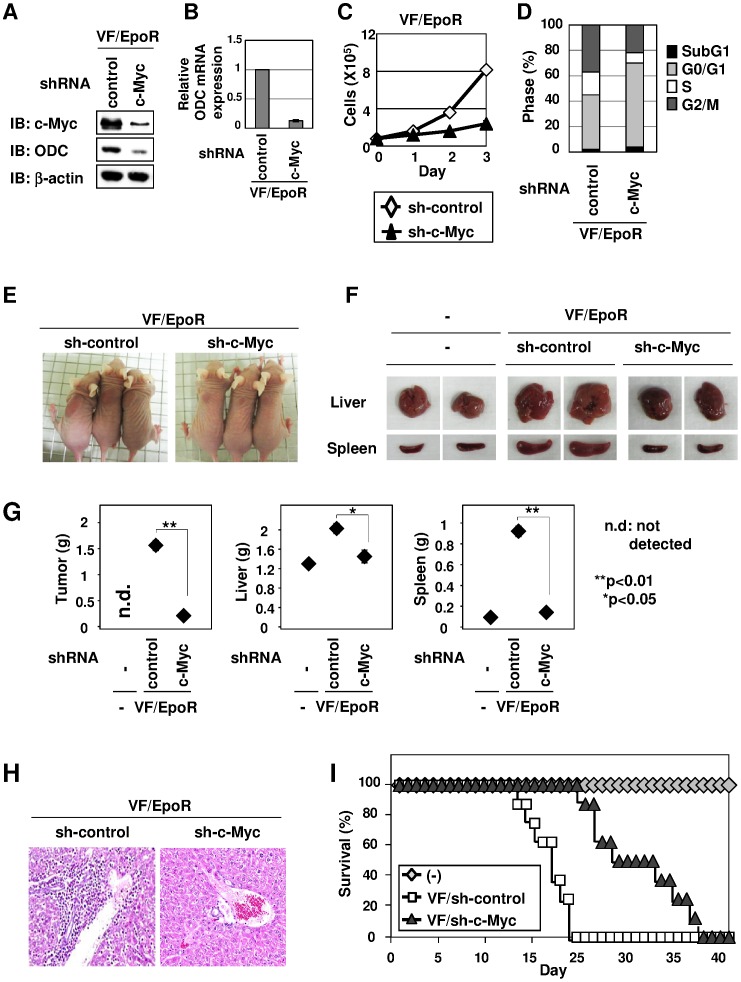
JAK2 (V617F)-induced cell proliferation and transformation require the expression of c-Myc. VF/EpoR cells were infected with retrovirus harboring shRNA against firefly luciferase (control) or c-Myc. (A) Transduced VF/EpoR cells were cultured without Epo for 12 hr. Whole cell lysates were immunoblotted (IB) with anti-c-Myc antibody, anti-ODC antibody or anti-β-actin antibody. (B) ODC mRNA was analyzed by quantitative real-time PCR. Data are the mean ± S.D. of the relative expression levels in three independent experiments. (C) Transduced VF/EpoR cells were cultured without Epo for 3 days. Viable cells were counted and shown in the graph. Results are the mean ± S.D. of three independent experiments. (D) Transduced VF/EpoR cells were cultured without Epo for 24 hr. Cells were then fixed, treated with propidium iodide (PI) and subjected to FACS analysis. (E-I) Transduced VF/EpoR cells were s.c. injected into nude mice (1×10^7^ cells/mice). (E) Nude mice were photographed 16 days post-inoculation. Arrows indicate tumors in nude mice. (F, G) Sixteen days post-inoculation, three mice were sacrificed. Morphological changes of the spleen and liver are shown in the photograph. The weights of the tumor, liver, and spleen were measured and plotted on the graph. * and ** indicate significant differences p<0.05 and p<0.01, respectively. (H) Sixteen days after inoculation, H&E staining was performed (magnification: ×100). (I) Mouse survival was monitored daily for 30 days post-inoculation (n = 10).

To address the role of c-Myc, we investigated whether subcutaneous (s.c) inoculation of transduced Ba/F3 cells into nude mice could induce tumor formation as well as VF/EpoR cells. While no tumor formation was exhibited in nude mice inoculated with control cells or In373 mutant cells, marked tumor formation was observed at the injected site of nude mice receiving T58A cells. Wild-type c-Myc also induced tumor formation, although the tumor was smaller than that caused by T58A mutant. The grade of subcutaneous tumor growth by c-Myc and T58A was comparable to when inoculated with VF/EpoR cells (Fig. 4A, C). However, whereas the liver and spleen were abnormally swollen when VF/EpoR cells were inoculated, inoculation with cells expressing c-Myc or T58A mutant failed to alter the volume or weight of these organs (Fig. 4B, C). In addition, H&E sections of the spleen and liver showed no obvious abnormalities in all types of mice (Fig. 4D). Furthermore, while most nude mice inoculated with VF/EpoR cells died within about 3 weeks (data not shown; see the reproducible and similar results of VF/sh-control in Fig. 5I), nude mice transplanted with cells transformed by wild-type c-Myc and T58A mutant did not exhibit lethality 14 weeks later, and this was completely different from the results of mice inoculated with VF/EpoR cells (Fig. 4E). Therefore, our observations clearly indicated that T58A mutant is not able to induce tumorigenesis as well as JAK2 (V617F) in spite of its growth factor-independent cell proliferation ability.

### JAK2 (V617F)-induced Cell Proliferation and Transformation Require c-Myc

To elucidate the role of c-Myc in the oncogenic functions of JAK2 (V617F), retroviruses harboring shRNA against c-Myc were used to silence the expression of endogenous c-Myc in VF/EpoR cells. Strikingly, we also succeeded in observing the reduction of ODC expression in both protein and mRNA (Fig. 5A, B). When c-Myc was knocked down, the proliferation of VF/EpoR cells was markedly decelerated (Fig. 5C). Next, we analyzed the alteration of the cell cycle by knock down of c-Myc. In VF/EpoR cells, knock down of c-Myc increased the proportion of cells in the G0/G1 phase, and oppositely reduced the S and G2/M phases (Fig. 5D). To test the requirement of c-Myc for tumorigenesis caused by JAK2 (V617F), we transplanted nude mice with VF/EpoR/sh-c-Myc cells. Sixteen days after inoculation, compared with mice inoculated with VF/EpoR/sh-control cells, the volume and weight of subcutaneous tumors were significantly reduced in mice inoculated with VF/EpoR/sh-c-Myc cells (Fig. 5E, G). Furthermore, knock down of c-Myc markedly attenuated the enlargement of the liver and spleen induced by JAK2 (V617F) (Fig. 5F, G). In the previous study [9], we observed the presence of GFP-positive cells, which must have derived from transplanted JAK2 (V617F)-positive cells in hepatocytes and splenocytes, suggesting that the JAK2 (V617F)-induced transformation of Ba/F3 cells finally tends to result in the infiltration of tumor cells into these organs. Strikingly, marked invasion of tumor cells into the liver was also abrogated in mice receiving VF/EpoR/sh-c-Myc cells (Fig. 5H). Furthermore, knock down of c-Myc effectively prolonged the life span of inoculated nude mice (Fig. 5I). These results clearly indicate that c-Myc is a critical mediator in JAK2 (V617F)-induced tumorigenesis *in vivo*.

### ODC Inhibitor Induces G0/G1 Arrest of Ba/F3 Cells Expressing JAK2 (V617F)

**Figure 6:**
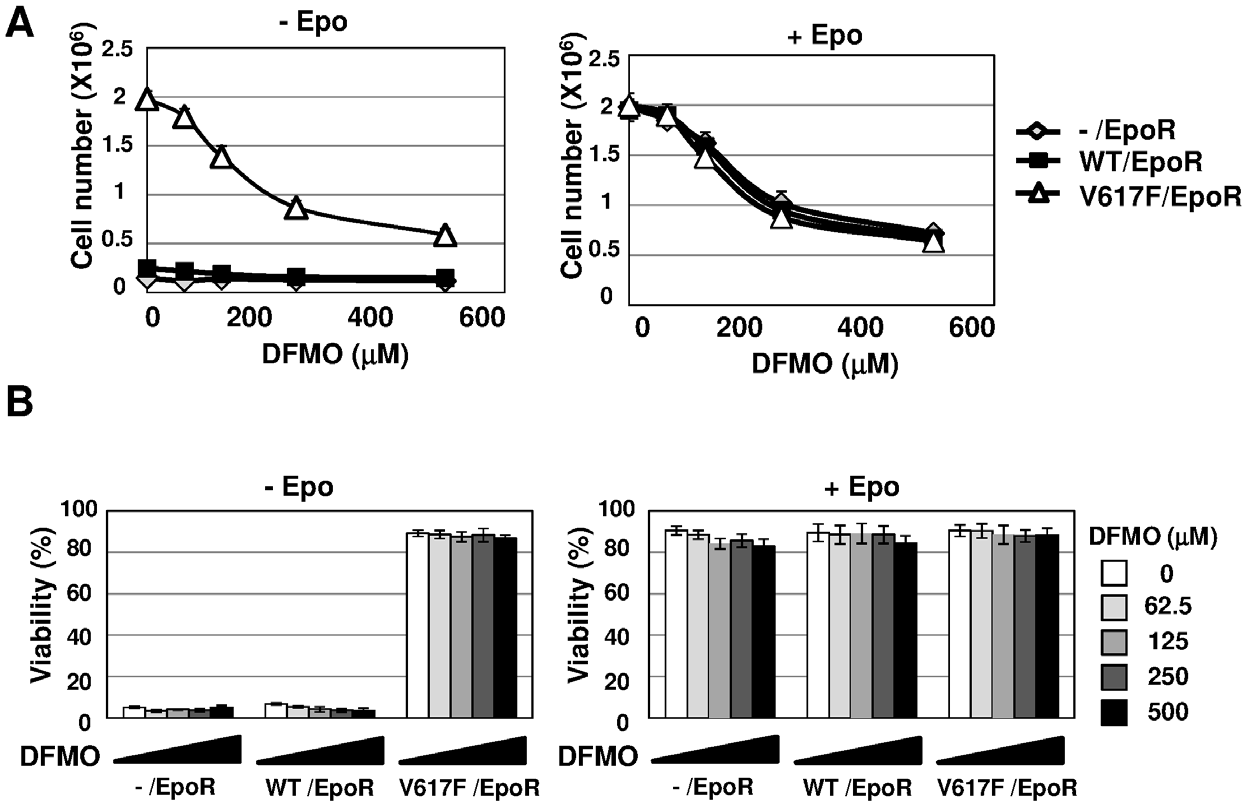
ODC inhibitor, DFMO, significantly induced G0/G1 arrest of transformed Ba/F3 cells by JAK2 (V617F). (A, B) EpoR cells, WT/EpoR cells and VF/EpoR cells were treated with the indicated concentrations of DFMO in the presence or absence of Epo (5 U/mL) for 3 days. (A) Viable cells were counted and shown in the graph. Results are the mean ± S.D. of three independent experiments. (B) The viability of these cells was evaluated by the trypan blue exclusion method. Results are the mean ± S.D. of three independent experiments.

Previous reports suggested that a target gene of c-Myc, ODC, plays a critical role in c-Myc functions, including cell proliferation and tumorigenesis [20], [21]; therefore, we next tested the effect of an ODC inhibitor, difluromethylornithine (DFMO), on the cell proliferation of EpoR cells, WT/EpoR cells and VF/EpoR cells [31], [32]. When cells are cultured in the presence of Epo, DFMO reduced the cell proliferation of EpoR cells, WT/EpoR cells and VF/EpoR cells in a dose-dependent manner. In the absence of Epo stimulation, only VF/EpoR cells proliferated and DFMO also inhibited its proliferation (Fig. 6A). However, DFMO failed to affect the viability of these cells (Fig. 6B). These results indicate that DFMO suppresses the cell proliferation mediating both of Epo-dependent mechanism and Epo-independent mechanism through JAK2 (V617F), and these effects do not require the induction of cell death.

**Figure 7:**
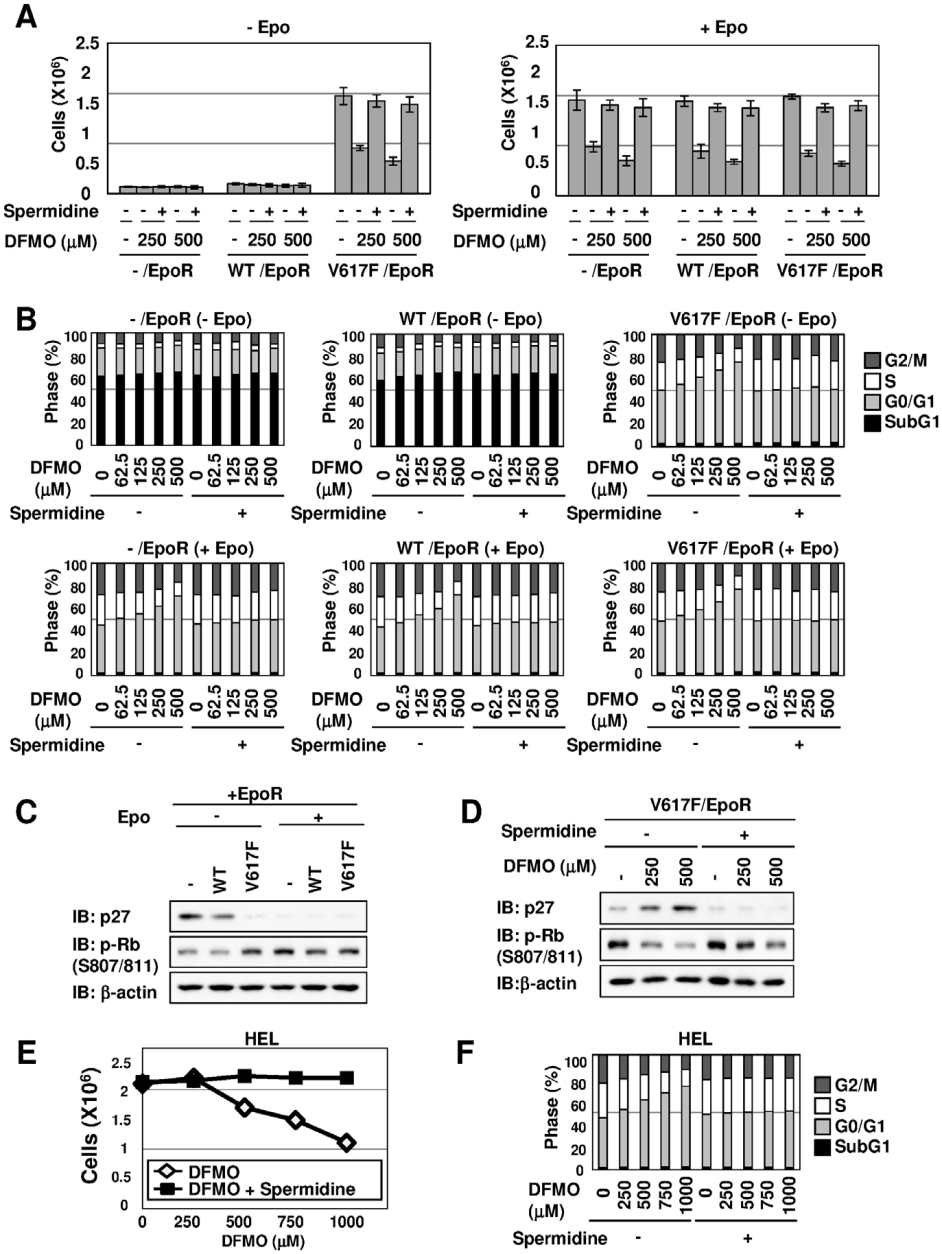
Spermidine abolished DFMO-induced G0/G1 arrest of transformed Ba/F3 cells by JAK2 (V617F). (A, B) EpoR cells, WT/EpoR cells and VF/EpoR cells cultured without Epo were treated with indicated concentrations of DFMO in the presence or absence of spermidine (5 µM) for 3 days. (A) Viable cells were counted and shown in the graph. Results are the mean ± S.D. of three independent experiments. (B) Cells were then fixed, treated with propidium iodide (PI) and subjected to FACS analysis. (C) EpoR cells, WT/EpoR cells and VF/EpoR cells were cultured in the presence or absence of Epo (5 U/mL) for 12 hr. Whole cell lysates were analyzed by immunoblotting (IB) with anti-p27 antibody, anti-phospho-Rb antibody (S807/811) or anti-β-actin antibody. (D) VF/EpoR cells cultured without Epo were treated with the indicated concentration of DFMO in the presence or absence of spermidine (5 µM) for 3 days. Whole cell lysates were immunoblotted (IB) with anti-p27 antibody, anti-phospho-Rb antibody (S807/811) or anti-β-actin antibody. (E, F) HEL cells were treated with the indicated concentrations of DFMO in the presence or absence of spermidine (5 µM) for 3 days. (E) Viable cells were counted and the results are shown in the graph. Results are the mean ± S.D. of three independent experiments. (F) Cells were fixed, treated with propidium iodide (PI) and subjected to FACS analysis.

Spermidine is produced from ornithine through a sequential reaction catalyzed by ODC and aminopropyl-transferases [17], [33]. Next, we tested whether spermidine canceled the DFMO-induced inhibition of cell proliferation. Strikingly, spermidine completely abolished the DFMO-induced cell proliferation arrest of VF/EpoR cells in the absence of Epo stimulation. Spermidine also counteracted the DFMO-induced inhibitory effect on the proliferation of EpoR cells, WT/EpoR cells and VF/EpoR cells, when stimulated with Epo (Fig. 7A). Furthermore, the Epo removal-induced accumulation of sub-G_1_ phase in EpoR cells and WT/EpoR cells was not affected with DFMO and/or spermidine. Interestingly, in VF/EpoR cells, DFMO increased the proportion of the cell cycle in the G_0_/G_1_ phase, and reduced the populations of S and G_2_/M phases. In contrast, spermidine clearly abolished the DFMO-induced alterations of cell cycle in VF/EpoR cells regardless of Epo stimulation. DFMO also caused G_0_/G_1_ arrest of Epo-stimulated EpoR cells and WT/EpoR cells, and spermidine counteracted the effect of DFMO (Fig. 7B). When EpoR cells and WT/EpoR cells stopped their proliferation by withdrawing Epo, the protein expression of p27^kip1^, an inhibitor of cyclin-dependent protein kinase 4/6 (CDK4/6), was increased (Fig. 7C), and this well correlated with the cell proliferative abilities. On the other hand, the enforced expression of JAK2 (V617F) reduced the expression of p27^kip1^, and this reduction was not affected by the presence or absence of Epo stimulation (Fig. 7C). The phosphorylation of retinoblastoma gene product (Rb) by CDK4/6 is an index to evaluate the S-phase entry during the cell cycle [34], [35]. As shown in Fig. 7C, the phosphorylation of Rb protein was only detected when stimulated with Epo, excepting the case of VF/EpoR cells, in which the Rb phosphorylation also could be detected in the absence of Epo stimulation. Interestingly, treatment with DFMO enhanced the expression of p27^kip1^ and reduced the phosphorylation of Rb in VF/EpoR cells as well as in EpoR cells and WT/EpoR cells in the absence of Epo (Fig. 7D). Strikingly, the addition of spermidine clearly abolished DFMO-induced p27^kip1^ expression and reduction of Rb phosphorylation, and these results are well reflected in the results shown in Fig. 7A and B. Furthermore, DFMO inhibited the proliferation of human HEL cells in a dose-dependent manner, and spermidine erased the inhibitory effect of DFMO on cell proliferation (Fig. 7E). Also in HEL cells, DFMO increased the proportion of cells in the G_0_/G_1_ phase, and oppositely reduced the S and G2/M phases; however, spermidine clearly canceled the alterations of the cell cycle caused by DFMO (Fig. 7F). Therefore, these results strongly suggest that the inhibition of ODC by DFMO caused the G_0_/G_1_ arrest of JAK2 (V617F)-mediated transformed cells, and this seems to be due to the induction of p27^kip1^ expression.

### ODC Inhibitor Abrogates JAK2 (V617F)-induced Tumorigenesis

**Figure 8:**
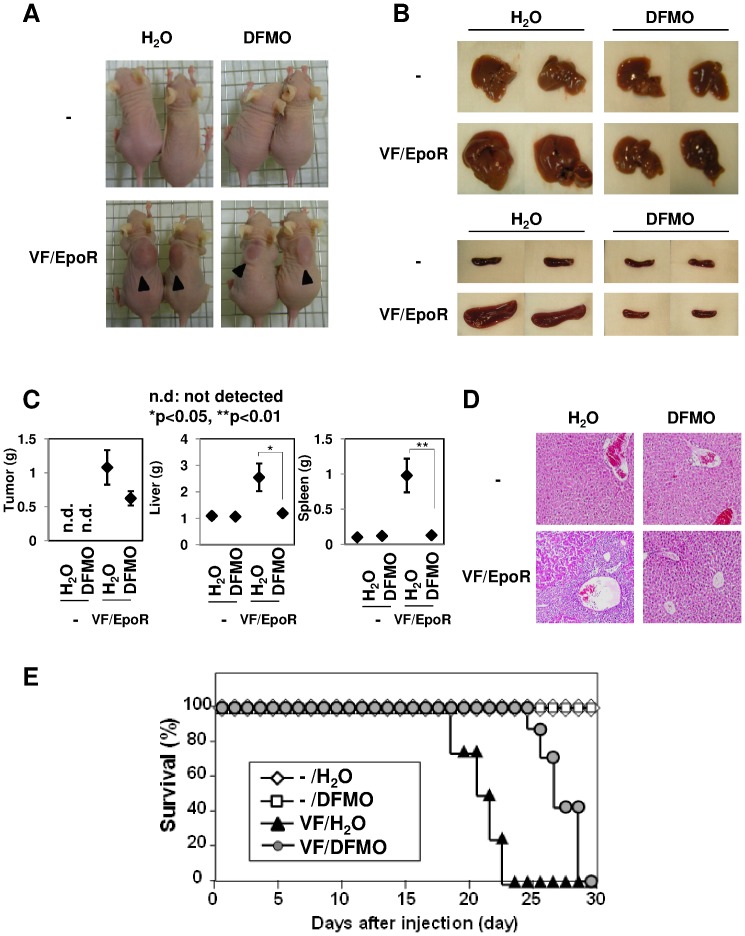
Treatment with ODC inhibitor, DFMO, significantly inhibited tumorigenesis induced by JAK2 (V617F). Ba/F3 cells were infected with empty virus (-) and retrovirus encoding JAK2 V617F mutant and EpoR. Transduced Ba/F3 cells were s.c. injected into nude mice (1×10^7^ cells/mice) and then given either standard water (H_2_O) or water containing 1% DFMO for 10 days. (A) Nude mice were photographed 16 days post-inoculation. Arrows indicate tumors in nude mice. (B, C) Sixteen days post-inoculation, three mice were sacrificed. Morphological changes of the spleen and liver are shown in the photograph. The weights of the tumor, liver, and spleen were measured and plotted in the graph. * and ** indicate significant differences p<0.05 and p<0.01, respectively. (D) Sixteen days after inoculation, sections of the liver were stained with H&E (magnification: ×100). (E) Mouse survival was monitored daily for 30 days post-inoculation (n = 10).

The current observations of the effect of DFMO on VF/EpoR cells (Fig. 7) strongly encouraged us to test the effect of DFMO on JAK2 (V617F)-induced tumorigenesis. As shown in Fig. 8A, the administration of 1% DFMO markedly delayed subcutaneous tumor formation derived from VF/EpoR cells, compared to in mice provided with normal drinking water (Fig. 8A). Indeed, tumor weight was obviously decreased by DFMO treatment (Fig. 8C). Sixteen days after inoculation, abrogation of the enlargement of the liver and spleen was observed in DFMO-treated mice transplanted with VF/EpoR cells (Fig. 8B). DFMO effectively suppressed the increase of the weight of the liver and spleen in mice inoculated with VF/EpoR cells (Fig. 8C). Moreover, in the liver of nude mice receiving VF/EpoR cells, the disruption of hepatocyte arrangement in lobules caused by infiltrated tumor cells disappeared by DFMO treatment (Fig. 8D). The life span of nude mice receiving VF/EpoR cells was also effectively extended by the administration of DFMO as compared with normal drinking water (Fig. 8E). Remarkably, in mice transplanted with control cells, DFMO induced no alteration of their appearance (Fig. 8A, upper photographs), the conditions of the liver and spleen (Fig. 8B, C and D), and life span (Fig. 8E), suggesting that the effect of DFMO is somehow specific to tumor cells. These observations clearly pointed out the crucial role of ODC in JAK2 V617F mutant-induced tumorigenesis and the effectiveness of DFMO for the treatment of MPNs.

## Discussion

We previously demonstrated that STAT5 plays key roles in JAK2 (V617F)-induced cellular transformation [10], and this suggested that the target genes of STAT5 may exhibit critical means for JAK2 (V617F)-induced tumorigenesis, including MPNs. In this study, we found that c-Myc was induced by JAK2 (V617F) through STAT5 activation. Theophile et al. also reported that c-Myc was highly expressed in bone marrow cells and accumulated in the nucleus of megakaryocytes in patients with MPNs than in healthy samples [36]; however, the mechanism of STAT5-induced c-Myc expression is still unclear. Basham et al. previously reported that they failed to identify STAT5 binding within the proximal c-Myc promoter *in vivo* [37], suggesting that the STAT5 responsive element might be located elsewhere. In addition, it has also been reported that c-Myc was regulated by AP-1 activation through ERK activation [38]. In another report, the activation of STAT5 was dispensable for JAK2-mediated c-Myc expression in the IL-3 signaling pathway [39]. Currently, we have no appropriate explanation of how STAT5 induces the expression of c-Myc, and this is a problem to be elucidated in future study.

JAK2 (V617F) also enhanced the protein stability of c-Myc by Akt-mediated inhibition of GSK3β (Fig. 3F). Once phosphorylated at Thr58 by GSK3β, c-Myc is recognized by Fbw7, a component of the SCF (Fbw7) ubiquitin ligase that is responsible for directing proteasome-mediated degradation [40], [41]. In addition, phosphorylation of EpoR at Y479 was necessary for JAK2 (V617F)-induced activation of Akt [29] and phosphorylation of GSK-3β (Fig. 3F). Our current observation showed that the mutation of EpoR (Y479F) significantly reduced the expression of c-Myc protein, whereas the expression level of c-Myc mRNA was not changed. Recently, a scaffold protein, Axin1, was shown to promote the degradation of c-Myc by coordinating complex formation, including c-Myc and GSK-3β [42], [43]. It was also noteworthy that the expression of Axin1 mRNA was reduced in breast cancer, and this reduction accelerated the oncogenic activity of c-Myc [42], [43]; however, our current analysis of the DNA array showed that alteration of the expression level of Axin1 mRNA was not found in JAK2 (V617)-induced transformation (data not shown). Therefore, Axin1 is most unlikely to be involved in c-Myc induction by JAK2 (V617F).

In the current study, we showed that the ODC is critical for JAK2 (V617F)-induced transformation. As reported previously, ODC is a target gene of c-Myc, and has a critical role in c-Myc-induced tumorigenesis [17], [32]. JAK2 (V617F) stimulates the activation of various signaling pathways, and it is a simple question whether the inhibition of ODC is also effective for the treatment of MPNs. The results from our current study clearly showed that Myc-ODC axis plays an essential role in the onset of MPNs. Strikingly, the previous study reported that a high concentration of polyamines was detected in blood plasma from *PV* patients [44], and this supports our working hypothesis that the c-Myc-ODC axis seems to play a critical role in episodes of *PV*. However, since this previous research was reported before the discovery of JAK2 mutation (V617F) in patients with MPNs, the relationship between polyamine production and JAK2 (V617F) has not been addressed. Therefore, it is necessary to analyze the existence of JAK2 mutation (V617F) in patients with MPNs and to measure polyamines in patients’ serum in order to confirm our model supporting the critical role of Myc-ODC axis in JAK2 (V617F)-induced tumorigenesis.

As shown in Fig. 7, treatment with DFMO caused G1 arrest and the accumulation of CDK inhibitor p27^kip1^ in JAK2 (V617F)-transformed cells, as in previous reports describing other types of tumors [45], [46]. Furuhata et al. also suggested that JAK2 (V617F) decreases the expression of p27^kip1^ [47]. Several tumor cells display the up-regulation of ODC, and enforced expression of ODC alone induces the tumorigenic transformation of fibroblasts [48]. In cells transformed by ODC, p27^kip1^ disappeared from the protein complexes, including cyclin E and CDK2 [49]. These events caused by ODC may contribute to the mechanism of JAK2 (V617F)-induced transformation.

Wernig et al. also reported that c-Myc was induced by JAK2 (V617F) in Ba/F3 cells and that c-Myc is important for the growth of Ba/F3 cells [11]. Furthermore, we clarified that the expression of c-Myc protein is regulated by JAK2 (V617F) mediating not only transcriptional, but also post-translational mechanisms. We previously demonstrated that the activation of Akt is essential for JAK2 V617F mutant to exhibit its transforming ability [29]. The activation of Akt prevents the phosphorylation of c-Myc at T58, and this seems to cause the up-regulation of c-Myc protein. In our study, the transplantation of Ba/F3 cells expressing c-Myc (T58A) into nude mice caused subcutaneous tumor formation; however, it failed to exhibit the same malignancy as cells expressing JAK2 (V617F). Combining this with our previous report that STAT5 has comparable tumorigenesis activity to JAK2 (V617F), it is strongly suggested that other target genes of STAT5 are required to exhibit metastatic ability. In future experiments, the identification of these target genes of STAT5 included in tumor malignancy will be important and studies are about to get underway. Although we used Ba/F3 cells to analyze the transforming activity of JAK2 (V617F) in this study, we will need to perform the experiments under more suitable conditions, such as by using a bone marrow transplantation assay in the future.

Next, we may need to confirm the therapeutic specificity of DFMO, since the concentrations of DFMO used in our study were high (200 to 600 µM). The effects of DFMO were canceled by the addition of spermidine; therefore, it is thought that the observed effects of DFMO are due to the inhibition of ODC (Fig. 7). In normal cells, the c-Myc-ODC signaling axis is moderately activated by growth factors such as cytokines. It is thought that this may explain why DFMO also affected the proliferation of normal cells, as shown in Fig. 6 and 7. Recently, the combination of several compounds including DFMO has been tested for the treatment and prevention of tumorigenesis, and it was reported that the risk of colorectal-adenoma recurrence could be reduced by 95% at maximum by using NSAIDs, Sulindac and DFMO together [50]. Also, our current observation indicates that the c-Myc-ODC axis has an essential role in JAK2 (V617F)-induced tumorigenesis, and suggests that DFMO would also be useful as an anti-cancer drug for MPNs.
